# Chronic Diarrhea Due to Eosinophilic Enterocolitis With Coexisting Eosinophilic Esophagitis

**DOI:** 10.7759/cureus.42349

**Published:** 2023-07-24

**Authors:** Ramesh R Avula, Ghanashyam Gangu, Abhishek SY, Vamsi D Yadav, LRS Girinadh

**Affiliations:** 1 Medical Gastroenterology, Andhra Medical College, Visakhapatnam, IND

**Keywords:** esophagitis, enterocolitis, steroids, eosinophilic gastrointestinal disorders, eosinophilic gastroenteritis

## Abstract

Eosinophilic gastrointestinal disorders (EGIDs) are a spectrum of disorders including eosinophilic esophagitis, eosinophilic gastroenteritis, and eosinophilic colitis. We report a case of EGID involving the esophagus, small intestine, and large intestine simultaneously. A 38-year-old male patient presented with chronic diarrhea, abdominal pain, and unquantified weight loss for the last two months, which not improving with routine empirical treatment. Endoscopy revealed erosions in the stomach, duodenum, terminal ileum, and proximal colon. Biopsy revealed eosinophilic infiltration in the esophagus, terminal ileum, and proximal colon. Contrast-enhanced CT showed multiple skip areas of short- and long-segment circumferential mural thickening with enhancement in the jejunum and ileal loops, causing mild luminal narrowing with pelvic ascites, indicating involvement of muscular and probably serosal layer to a lesser degree (absence of obstructive symptoms with minimal ascites) along with predominant mucosal involvement (responsible for clinical symptoms). The patient was treated with elimination diet, systemic corticosteroids, and montelukast. Diarrheal episodes decreased, and the treatment was shifted to oral budesonide. We believe it to be one of the first reports to show a simultaneous involvement of the esophagus, small intestine, and large intestine, along with mucosal and mural involvement. It strengthens the fact that a common underlying pathogenesis causes EGIDs and an underlying muscular layer involvement in patients with predominant mucosal disease.

## Introduction

Eosinophilic gastrointestinal disorders (EGIDs) are defined as disorders that primarily affect the gastrointestinal (GI) tract with eosinophil-rich inflammation in the absence of known causes for eosinophilia (e.g., drug reactions parasitic infections, malignancy). They include eosinophilic esophagitis (EOE), eosinophilic gastroenteritis (EGE), and eosinophilic colitis (EC). EOE is the commonest among EGIDs, followed by EGE and EC with an estimated prevalence of 39-56.7, 6.4, and 3.5 cases per 100,000, respectively. Though the incidence of EGIDs has been increasing from the 1990s, they remain a rare entity overall, making it difficult to conduct research and investigate therapeutics. Clinical decisions usually rely on data from case reports and case series. EGIDs, though presenting with different symptoms based on the site of involvement, are thought of having a common pathogenesis with local factors affecting the severity of the disease. This is strengthened by the fact that around 10% of EGE and EC patients have coexisting EOE. EGID involving the esophagus, small intestine, and large intestine simultaneously are rare especially considering that EC is extremely rare. We intend to report a case of EGID involving the esophagus, small intestine, and large intestine simultaneously.

This case report (abstract) has been presented as a poster at the Annual Conference of the Indian Society of Gastroenterology (ISGCON) 2022 conference held in Jaipur, India.

## Case presentation

A 38-year-old male patient presented with chronic diarrhea (large volume, three to four days, no blood in stool), abdominal pain (diffuse, more near umbilicus), and unintentional unquantified weight loss for last two months, which was not improving with routine empirical treatment with antibiotics, anti-protozoals, and anti-diarrheals. Stool test did not reveal any ova or cysts. He did not have any recent travel history or any past history of asthma/allergies. No family history of similar symptoms was reported. Viral markers were negative (Table 1 ).

**Table 1:** Laboratory data HIV, human immunodeficiency virus; HBsAg, hepatitis B surface antigen; anti HCV, anti-hepatitis C virus antibodies

Parameter (units)	Test report (reference values)
Hemoglobin (gm/dL)	14.3 (males: 14-16; females: 12-16)
Total leukocyte count (cells/microliter)	24,200 (neutrophils: 81%; lymphocytes: 10%; eosinophils: 2%) (4,000-11,000)
Platelet count (lakhs/microliter)	3.69 (1.5-4.5)
Total bilirubin (mg%)	1.2 (<1.5)
Direct bilirubin (mg%)	0.5 (<1)
Alanine transaminase (IU/L)	25 (males: <29, females: <19)
Aspartate transaminase (IU/L)	22 (males: <40; females: <32)
Alkaline phosphatase (IU/L)	262 (100-320)
Serum protein (gm%)	6.5 (6-8.3)
Serum albumin (gm%)	3.8 (3.4-4.4)
Viral markers (HIV, HBsAg, anti-HCV)	Non-reactive
Stool for ova and cyst	Negative

**Figure 1:**
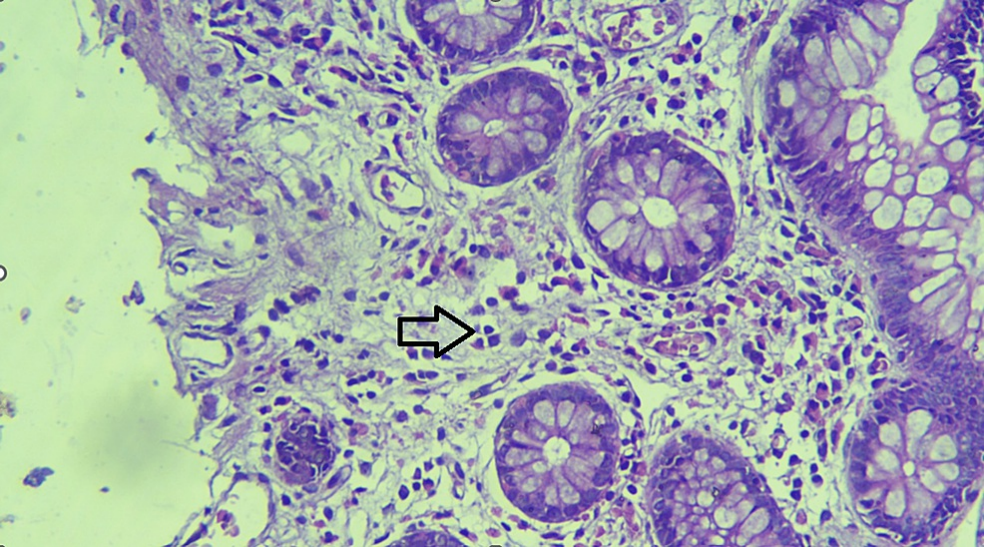
Ileal biopsy showing prominent eosinophils seen in the epithelium and stroma

**Figure 2:**
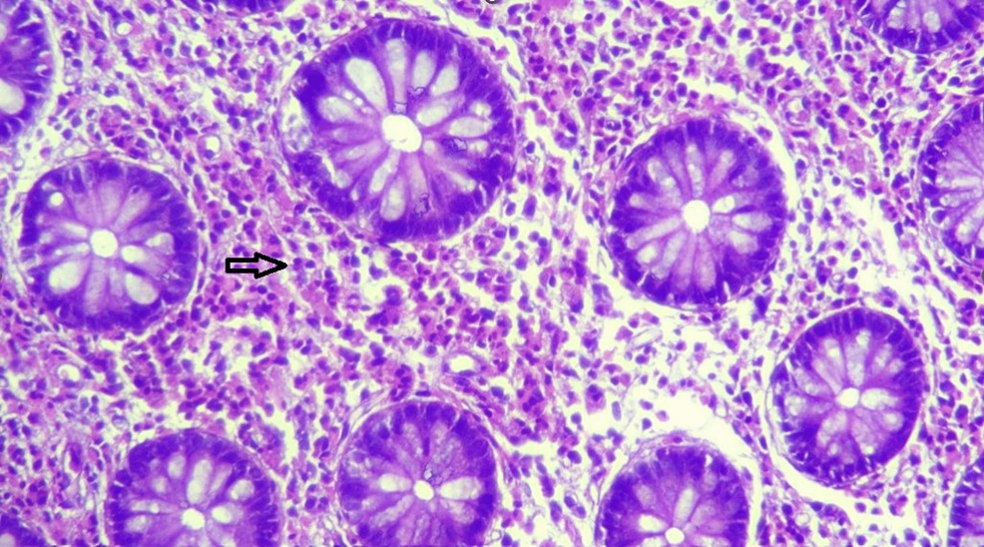
Ascending colon showing diffuse sheets of eosinophils

**Figure 3:**
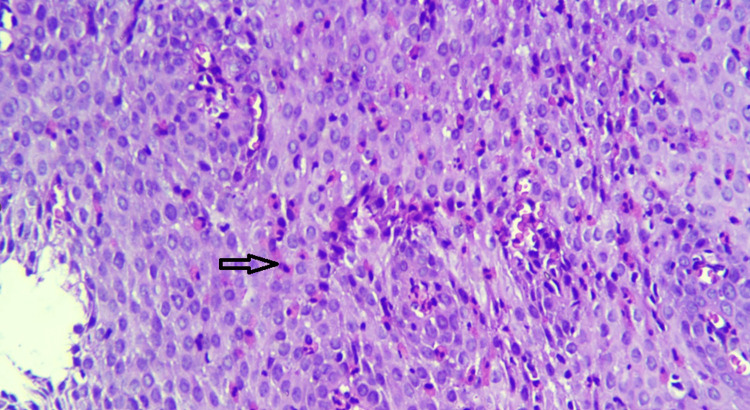
Esophageal biopsy showing prominent eosinophils seen in the epithelium and stroma

Endoscopy (upper and lower GI) revealed erosions in the stomach, duodenum, terminal ileum, and proximal colon. Biopsy revealed eosinophilic infiltration in the terminal ileum and proximal colon (>100/HPF) (Figures 1, 2). Subsequent esophageal biopsy showed intense eosinophilic infiltration of mucosa (>100/HPF) (Figure 3). Gastric and duodenal mucosa showed nonspecific inflammation. Peripheral blood did not show any eosinophilia.

Contrast-enhanced CT showed multiple skip areas of short- and long-segment circumferential mural thickening with enhancement in the jejunum and ileal loops in the upper mid and lower abdomen, causing mild luminal narrowing with pelvic ascites, indicating involvement of muscular and probably serosal layer to a lesser degree (absence of obstructive symptoms with minimal ascites) along with predominant mucosal involvement (responsible for clinical symptoms).

The patient was treated with elimination diet, systemic corticosteroids (prednisolone 40 mg once a day), and montelukast (10 mg once a day). He responded well, diarrheal episodes and abdominal pain decreased; after one week, the treatment shifted to oral budesonide, which was continued for four weeks. Montelukast was continued. The patient started regaining weight.

## Discussion

There are scant data on simultaneous involvement of different segments of the gut in EGIDs [1,2]. Most commonly, the esophagus has been shown to be involved in EGE and EC [3]. We believe that this is one of the first reports to show a simultaneous involvement of the esophagus, small intestine, and large intestine, along with mucosal and mural involvement. Lack of eosinophilic infiltration in the duodenal and gastric mucosa was also peculiar considering the fact that they are the most common organs to be involved in EGE.

EGIDs have a male predominance, with patients usually presenting in the second to fourth decade of life. Esophageal mucosa does not have eosinophils normally, unlike in the stomach and intestine, where eosinophils are present in small numbers. Peak eosinophil counts of 30/HPF in the stomach and 50/HPF in the duodenum have been proposed for the diagnosis of eosinophilic gastritis and duodenitis, respectively [4].

EGE has three subtypes based on the involved layer of intestine: mucosal, muscular, and serosal types [5]. The prevalence of each subtype is not known. Our patient had mucosal involvement and indirect evidence of muscular layer involvement, as indicated by radiologic findings of segmental mural thickening with mild narrowing of the small intestine. The segmental nature of mural involvement also indicates patchy nature of infiltration, which makes it necessary to take multiple biopsies to avoid false-negatives in mild cases. Mild ascites also suggests possible involvement of serosa. Our patient’s symptoms were attributed to predominant small intestinal mucosal involvement.

Macroscopically, the patient had erosions of involved organs. Polyps/strictures were absent. The patient did not have any dysphagia though biopsy showed esophageal involvement. He did not have any history of allergies/asthma. No other organ system was involved. Intestinal biopsies did not show any granulomas. Comprehensive allergic tests failed to reveal any allergies.

Treatment options for EGIDs include elimination diet, elemental diet, oral/topical/systemic steroids, montelukast, and IL-5 antibodies such as reslizumab; our case showed that budesonide can be used successfully to treat intestinal involvement in EGIDs [6]. Surgery might be required in those with obstruction due to muscle layer involvement [7].

## Conclusions

The present case highlights simultaneous involvement of the esophagus, small intestine, and large intestine, indicating a common underlying pathogenesis for EGIDs. The symptom complex is often dominated by a particular segment, i.e., its phenotypic expression is modified by as yet unknown genetic or dietary factors. This case also points to a possible subclinical involvement of muscular/serosal layers in many cases. In addition, oral budesonide preparation was successful in alleviating the symptoms of the patient with minimal systemic adverse reactions. It also reiterates the important role of endoscopy in the diagnostic work-up of chronic diarrhea.
